# Snow Melting Performance of Graphene Composite Conductive Concrete in Severe Cold Environment

**DOI:** 10.3390/ma14216715

**Published:** 2021-11-08

**Authors:** Xinjie Wang, Yongkang Wu, Pinghua Zhu, Tao Ning

**Affiliations:** Department of Civil Engineering, Changzhou University, Changzhou 213164, China; cczuwyk@163.com (Y.W.); zph@cczu.edu.cn (P.Z.); 15061960657@163.com (T.N.)

**Keywords:** graphene composite conductive concrete, graphene content, snowmelt test, electrode spacing, energizing voltage

## Abstract

The use of conductive concrete is an effective way to address snow and ice accretion on roads in cold regions because of its energy saving and high efficiency without interruption of traffic. Composite conductive concrete was prepared using graphene, carbon fiber, and steel fiber, and the optimum dosage of graphene was explored with resistivity as the criterion. Subsequently, under the conditions of an initial temperature of −15 °C and a wind speed of 20 km/h, the extremely severe snow event environment in cold regions was simulated. The effects of electrode spacing and electric voltage on snow melting performance of conductive concrete slab were explored. Results showed that graphene can significantly improve the conductivity of conductive concrete; the optimal content of graphene was 0.4% of cement mass in terms of resistivity. The snow-melting power of conductive concrete slab decreased with increase in electrode spacing and increased with increase in on-voltage. For an optimal input voltage of 156 V and an optimal electrode spacing of 10 cm, the time required to melt a 24 h snow thickness (21 cm), accumulated during a simulated severe snow event, was only 2 h, which provides an empirical basis for the application of graphene composite conductive concrete to pavement snow melting in cold regions.

## 1. Introduction

Severe ice and snow events are climate events that affect traffic safety and the service reliability of concrete road engineering. In recent years, the frequency of severe ice and snow events has increased year-on-year, which has not only seriously disrupted normal urban activities, but has also caused great loss of property and life. Statistical evidence suggests that approximately 10–15% of road traffic accidents are caused by severe ice and snow events [1] which presents severe challenges to the sustainable development of the social economy. The timely melting of snow is an effective measure to prevent accidents. Traditional snow melting methods include manual removal, mechanical removal, and chemical snow melting. However, the manual removal method can affect traffic and has limited efficiency and high cost. The mechanical removal method demonstrates high efficiency for snow and ice melting, but the utilization rate of equipment is low, and the maintenance cost is high. Use of chloride deicing agents in chemical melting is cheap and effective, but they have a corrosive effect on concrete and cause environmental pollution. Conductive concrete attracted wide attention as soon as it became available because of its timely snow melting, green environmental credentials, and because it does not require interruption of traffic.

Traditional conductive concrete pavements usually use graphite, carbon fiber, and steel fiber, as conductive fillers, but they have certain limitations. Adding graphite into concrete can significantly improve the conductivity of cementitious materials, but due to the large amount of graphite required, the mechanical properties of the materials decrease significantly, such that they cannot meet the basic strength requirements of road engineering [2,3]. Carbon fiber has a large aspect ratio, and readily forms a relatively stable conductive network in concrete, thereby enhancing the conductivity of concrete [4,5,6]. However, with increase in carbon fiber content it can agglomerate, which makes it difficult for concrete to form uniformly. Steel fiber can improve the conductivity of concrete [7,8,9], though it can gradually rust during conductive heating, resulting in a significant decline in conductivity, which is not conducive to its long-term use in conductive concrete [10,11,12]. Therefore, seeking conductive materials which have excellent electrical conductivity, strong corrosion resistance, and which meet mechanical property requirements, has become the focus of research into conductive concrete.

With the development of powder technology and nanotechnology, conductive materials are also being developed at the nanometer scale. Graphene is a new nanomaterial with excellent mechanical and electrical conductivity. Its resistivity is only 10^−6^ Ω·cm, which can enable conductive concrete to achieve good electrical conductivity at low concentration. It has potential application in conductive concrete [13,14,15,16,17]. Rehman et al. [18] added 0.03% multi-layer graphene to cement matrix composites and the resistivity of the cement mortar decreased by 67.8%. Bai et al. [19] found that when the content of graphene reached 1.2%, the resistivity of 28 day-aged graphene cement-based concrete could be 9.1 Ω·m. Current issues though include the price of graphene products which is still relatively high over the short term, and that, with high content levels, graphene is difficult to disperse uniformly, which affects the electrical conductivity. Summarizing the advantages and disadvantages of various conductive materials, Wu et al. [20] suggested that the use of multiphase conductive materials was beneficial in significantly improving the conductivity of conductive concrete. However, to avoid overly complex preparation processes, two or three types of conductive materials should be used, and conductive materials of complementary function and scale should be employed to give full effect to their conductive properties. Carbon fiber and steel fiber are easily connected to the conductive network in concrete, which can effectively reduce the content of graphene. Graphene can also significantly improve the conductivity and durability of conductive concrete and has good complementary characteristics.

In order to further explore the application of graphene in the design of cement-based materials, it is necessary to assess the feasibility of the application of graphene composite conductive concrete to road snow melting in severe cold regions, and to consider solutions to the existing problems of conductive concrete in conductivity, snow melting efficiency, and snow melting energy consumption. At present, conductive concrete slabs are prepared for submission to deicing and snow melting tests [21,22,23,24,25]. Sassani et al. [7] added 1% carbon fiber to concrete to prepare carbon fiber conductive concrete pavement with a size of 5.57 × 3.8 × 0.09 m^3^ and with electrode spacing of 1 m. At the Des Moines International Airport in the US, 38mm thick snow melted within 3.5 h of exposure to 210V alternating current outdoors. Arabzadeh et al. [26] added 1% carbon fiber to asphalt concrete to prepare conductive asphalt concrete slabs of size 380 × 210 × 75 mm^3^ with electrode spacing of 125 mm. They applied 40 V AC (alternating current) and melted 190 mm thick snow over 2 h. Tang et al. [27] prepared 400 × 400 × 40 mm^3^ carbon fiber conductive concrete plates and applied a 25 mm thick insulation layer at the bottom of the plates, which melted 31 mm thick snow in the field over 1 h. The above snow-melting tests have confirmed the snow-melting performance of conductive concrete slab, but there has not been further investigation into the factors influencing the conductive performance of conductive concrete. There is, moreover, a lack of targeted research on heating efficiency in the practical application of conductive concrete.

According to existing research, the layout parameters of conductive concrete slab electrodes have a great influence on the thermal efficiency of the material [28,29,30], but there are few studies on the factors relevant to electrode spacing in real-time snowmelt tests. Zhang et al. [31] used a finite element model to analyze the influence of spacing and the buried depth of carbon fiber heating lines on temperature uniformity. It was found that the temperature difference on the plate surface increased with increased spacing of the heating line and decreased with increase in buried depth. Reasonable parameters for the heating line were found to be a spacing ≤100 mm and a buried depth ≥50 mm. Wu et al. [32] concluded that when the electrode spacing of carbon fiber conductive concrete plate was 5–15 cm, the rate of temperature rise decreased with an increase in electrode spacing. Won et al. [33] found that the heating rate of the plate surface decreased with increase in electrode spacing in the conductive concrete prepared with metal materials. Malakooti et al. [34] studied the heating effect of 50.8 cm, 64.8 cm, and 91.43 cm electrode spacing on conductive concrete pavement, and found that the power density and temperature rise efficiency were the highest when the electrode spacing was 91.43 cm. In summary, the optimal electrode spacing of graphene composite conductive concrete plate is a key factor in improving the snow melting efficiency of conductive concrete and reducing later operation and maintenance costs.

Most studies on the snow melting and deicing behavior of conductive concrete have not considered the grade of the severe snow event, or the differences in the energy consumption of snow melting under different snow intensities. Currently, investigations are mainly focused on snow melting efficiency and required energy consumption by adjusting the input power, specifically, the input voltage [35]. Zhang et al. [36] showed that under 30 V direct current voltage, the temperature of the prepared conductive concrete could increase by 14–16 °C within 235 min. Li et al. [37] carried out a series of snow melting and deicing pavement investigations under climate conditions characteristic of China. It was found that when the temperature is −15 °C, the heating power should exceed 500 W/m^2^, when the temperature is −10 °C, the heating power should exceed 350 W/m^2^, and when the temperature is −5 °C, the heating power should exceed 250 W/m^2^. Therefore, it is necessary to optimize the voltage required for snow melting according to the specific snow event conditions, to improve the efficiency and economy of snow melting.

In this study, three-phase, composite conductive concrete was prepared with multilayer graphene, carbon fiber, and steel fiber as conductive materials, and its conductive mechanisms were studied. The optimal content of graphene in conductive concrete was determined by its resistivity. In addition, the effect of the electrode spacing arrangement and the electric voltage on snow melting efficiency of conductive concrete slab was explored, in order to meet the snow melting performance requirements of graphene composite conductive concrete under severe snow conditions.

## 2. Experimental

### 2.1. Raw Materials

P·O 42.5 Portland cement was used as cement. Natural river sand, with 3.0 fineness modulus, 0.1% mud content, and 2620 kg/m^3^ apparent density, was used as fine aggregate. Gravel of 5–16mm calibre, with 0.2% mud content, and 2587 kg/m^3^ apparent density, was used as coarse aggregate. Graphene used in this experiment was produced by Changzhou Sixth Element Materials Science and Technology Co., Ltd., Changzhou, China. Graphene, carbon fiber, and steel fiber were selected as conductive fillers; their performance parameters are shown in Table 1, Table 2 and Table 3, respectively.

In this paper, 0.4 wt.% carbon fiber and 1% vol. steel fiber were mixed into concrete according to the volume exclusion law of multiphase conductive materials. In the composite, 0.2, 0.4, 0.6, 0.8 and 1 wt.% of graphene were selected. Tributyl phosphate, with 0.03% dosage of cement, was used as a defoamer. Superplasticizer, with 2% dosage of cement, was used as a water reducer. Sodium dodecyl benzene sulfonate was used as the dispersant of graphene; the dosage was twice that of graphene. Sodium nitrite was selected as the conductive strengthening agent, and the dosage was 2% of cement. The proportion of graphene composite conductive concrete is shown in Table 4.

### 2.2. Sample Preparation

Firstly, the graphene dispersion was prepared. An electronic balance was used to weigh graphene and sodium dodecyl benzene sulfonate. Then the mixed powder was placed into a 500 mL beaker and water was added. The beaker was placed on a magnetic stirrer and stirred for 5 min at 250 r/min. When the mixture was uniformly dispersed, without obvious foam, the mixture was poured into several 50 mL flat-bottom centrifuge tubes, and the centrifuge tubes were placed in an ultrasonic dispersion instrument at 500 °C and 40 kHz for 30 min. Finally, the centrifuge tube was removed and cooled to room temperature to prepare the graphene dispersion.

For the preparation of the graphene composite conductive concrete test block and plate, cement, sand, stone, and steel fiber were first added to the cement mortar mixer and slowly stirred for 60–120 s. When the basic distribution of steel fiber was uniform, 3/4 of graphene dispersion was slowly added and stirred for 60 s. Carbon fiber and sodium nitrite were then added, and finally 1/4 of the graphene dispersion was added with polycarboxylate superplasticizer and tributyl phosphate with slow stirring for 120 s. When the mixture was evenly mixed and no obvious bubbles were generated, the mixture was poured into the mold to prepare the graphene composite conductive concrete test block and plate, the electrode plate was inserted, and the vibration table was used for compaction molding. After natural curing for 24 h, the block was demolded and put into a standard curing room for 28 days at 20 °C and 95% humidity.

**Figure 1:**
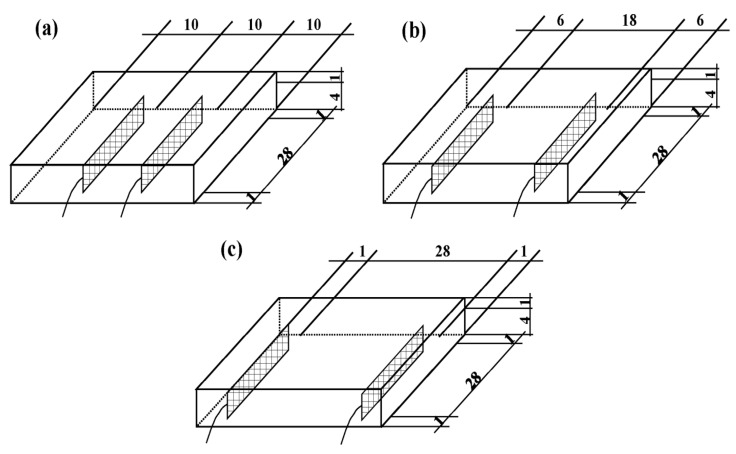
Graphene composite conductive concrete slabs with different electrode spacings. (**a**) 10 cm, (**b**) 18 cm, (**c**) 28 cm.

The size of the graphene composite conductive concrete slab was 30 × 30 × 5 cm^3^, the electrode size was 29 × 29 × 4 cm^3^, the electrode spacings were 10 cm, 18 cm, and 28 cm, and the on-voltages were 128, 156, and 220 V, respectively. The electrode layout is shown in Figure 1.

### 2.3. Measurement

#### 2.3.1. Compression Measure

The specimens of dimensions 100 × 100 × 100 mm^3^ were removed after standard curing for 28 d in the curing room. Applying the standard test method for the physical and mechanical properties of concrete (GB/T 50081 2019) [38], the compressive strength of the concrete was tested, using an electro-hydraulic universal servo testing machine, for graphene contents of 0, 0.2, 0.4, 0.6, 0.8, and 1.0 wt.%. The test value was accurate to 0.01 MPa.

#### 2.3.2. Electric-Conductivity Measure

The electrical conductivity of the graphene composite conductive concrete after 28 days of curing was characterized by assessment of resistivity. The resistivity of the graphene composite conductive concrete was measured by the two-electrode method, and the adjustable voltage regulated AC power supply (YM-1800 A) was used for testing. With the power off, the positive and negative poles of the concrete test block were connected to the positive and negative poles of the power supply by wire. The AC power was connected, and the output frequency of the AC power supply was adjusted to 50 HZ, with a voltage of 108 V. The voltage and current of the power instrument screen were read, and the resistivity was calculated according to the volt-ampere law and Equation (1).

(1) $$\rho =\frac{RS}{L}$$

where ρ, the resistivity, Ω·cm; R, resistance, Ω; S, contact area between electrode network and test block, m^2^; L, distance between electrode networks, m.

#### 2.3.3. Microscopic Analysis

After the specimens had been cured for 28 days, the interfacial transition zone of the graphene composite conductive concrete specimens, with graphene content of 0, 0.2, 0.4, 0.6, 0.8, and 1.0 wt.%, were sampled. After the samples were treated, they were examined by Zeiss SUPRA55 scanning electron microscope in low vacuum mode, and SEM scanning was performed under 20 kV accelerated voltage.

#### 2.3.4. Snowmelt Experiment

After the curing of the graphene multiphase conductive concrete slab was completed, it was removed. The AC voltage stabilizer was connected to the positive and negative electrodes of the board with a wire in the power-off state. A polyurethane foam plate with a thickness of 2 cm was laid around and at the bottom of the concrete plate as an insulation layer and placed in a refrigerator (BC/BD-307HNE) to effect an indoor snow melting test. The snow was evenly spread on the surface of the conductive concrete plate to the thickness of 21 cm (the thickness is equivalent to the 24 h snow volume when the ice and snow event grade is the grade of heavy rain and snow [39,40]). The ambient temperature of the refrigerator was adjusted to −15 °C, the fan gear was adjusted, and the wind speed was adjusted to 20 km/h.

**Figure 2:**
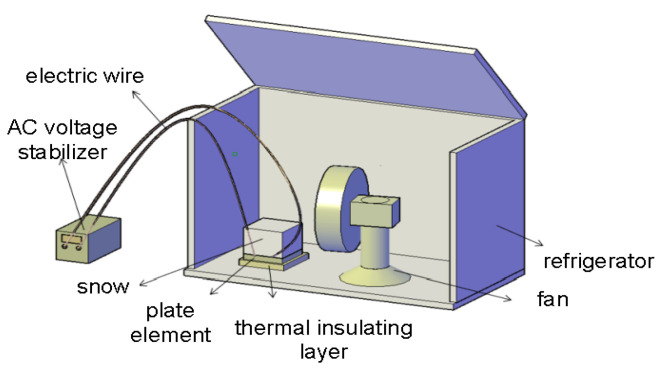
Operational picture of snowmelt test.

The AC voltage regulator (YM-1800 A) was used to input 108 V, 156 V, and 220 V AC currents. The snow thickness was measured and recorded every 15 min until complete melting was achieved. The current and voltage data of the AC voltage regulator were read and the resistance of the graphene composite conductive concrete plate was calculated according to ohmic law. An infrared thermometer (HT650C) was used to test and record the temperature of each measuring point distributed evenly on the surface of the concrete slab. Finally, the average value of the temperature measuring points was taken as the temperature of the concrete slab, and the time-average temperature diagram was drawn. The schematic diagram of the snow melting test is shown in Figure 2.

## 3. Results and Discussion

### 3.1. Compressive Strength

The results of the compressive tests are presented in Table 5. The compressive strength of concrete can be effectively improved by adding an appropriate amount of graphene into concrete. When the graphene content was 0–1.0% of the cement quality, the compressive strength of the graphene composite conductive concrete first increased and then decreased. When the content of graphene was 0.4% of the cement mass, the internal pores of concrete were effectively filled, and the compressive strength was 45.3 MPa, which was 6% higher than that without graphene. However, the effect of the content of graphene in concrete was not simply ‘the more the better’. When the content increased to 1%, the compressive strength was only 37.6 MPa, which was 11.6% lower than that without graphene. This is because graphene has a large specific surface area, and excessive graphene will absorb a large amount of water, resulting in incomplete hydration of the concrete, thus affecting its compressive strength. In summary, the optimal graphene content of the graphene composite conductive concrete based on compressive strength was 0.4%.

### 3.2. Effect of Graphene Content on Resistivity of Multiphase Conductive Concrete

**Figure 3:**
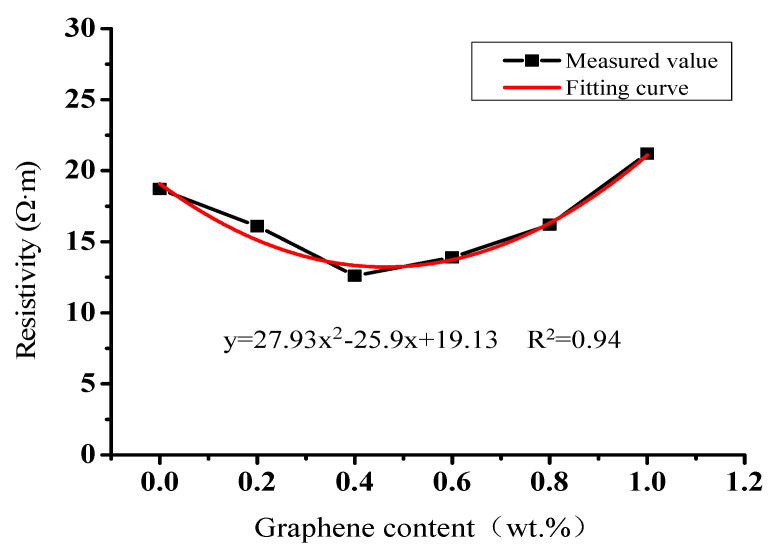
Relationship curve between graphene content and 28 d age resistivity of graphene composite conductive concrete.

The resistivity results of conductive concrete at 28 days with different graphene contents are shown in Figure 3. When a small amount of graphene was added at the beginning, the resistance of concrete showed a downward trend, because the resistivity of graphene itself was low. When graphene was added into concrete, the overall resistivity of the cementing material was reduced. When the content of graphene was close to 0.4 wt.%, conductive seepage occurred, manifested in the significant enhancement of the conductivity of concrete and the transformation from a bad conductor to a good conductor. The corresponding volume fraction or volume fraction interval is called the percolation threshold [41,42]. In addition, the electron mobility of graphene is more than 15,000 cm^2^/V·s at room temperature; adding an appropriate amount of graphene in concrete can reduce the potential energy of electronic transition in the concrete matrix. When inputting AC test resistance, the probability of electronic transition can be greatly improved, and then the conductivity of concrete can be improved.

However, with an increase of graphene content above 0.4 wt.%, the resistivity gradually stopped decreasing and showed an upward trend. With a large amount of graphene, it is easy for it to agglomerate and affect the formation of the conductive network. Furthermore, due to the large specific surface area, a large amount of water will be absorbed during the preparation of conductive concrete, which affects the conductivity. In order to meet the design resistivity for snowmelt and de-icing of the graphene composite conductive concrete, and to reduce the cost within the design range of resistivity, the optimal content of graphene was determined to be 0.4 wt.%.

### 3.3. Conductive Microscopic Mechanism Analysis under Different Graphene Contents

**Figure 4:**
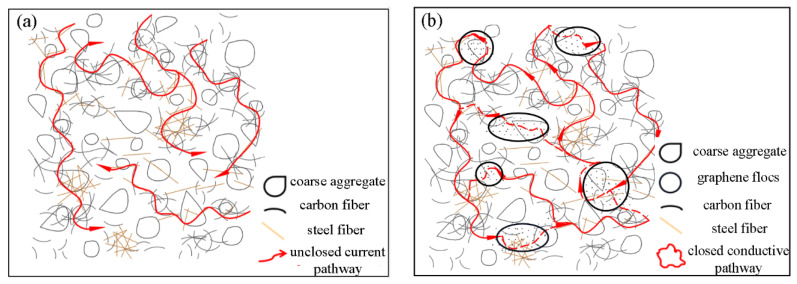
Diagram of conductive network of composite conductive concrete; (**a**) carbon fiber-steel fiber; (**b**) carbon fiber-steel fiber-graphene.

With the incorporation of graphene, the conductive network in the composite conductive concrete is shown in Figure 4. Before graphene was added, due to the large length to diameter ratio of the carbon and steel fiber, overlap to form the conductive network occurred readily, as shown in Figure 4a; however, the two fiber types are prone to become discrete when stirring, which inevitably leads to discontinuities and loops. When the graphene content increases to the percolation threshold, an appropriate amount of graphene can enhance the connectivity and dynamic closure of the fibrous conductive network through electronic transition, thus forming a three-phase composite conductive system in the concrete, as shown in Figure 4b. Due to the electron tunneling effect in the conductive concrete [43], the overall conductivity of graphene composite conductive concrete is significantly enhanced under the synergistic effect of the conductive network.

**Figure 5:**
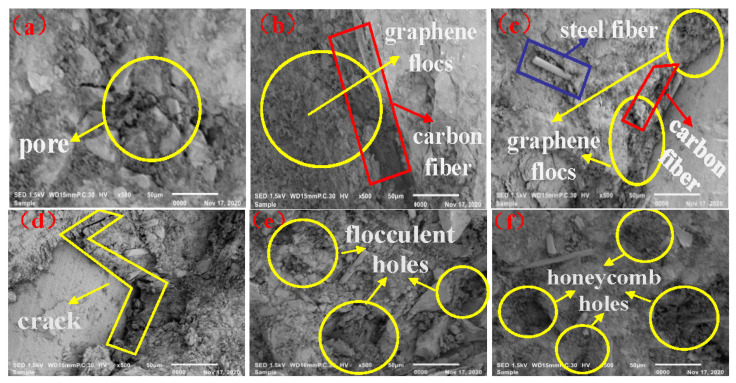
SEM test diagram of graphene composite conductive concrete with different graphene contents: (**a**) 0 wt.%, (**b**) 0.2 wt.%, (**c**) 0.4 wt.%, (**d**) 0.6 wt.%, (**e**) 0.8 wt.%, (**f**) 1.0 wt.%.

SEM images of the interface transition zone of the composite conductive concrete samples with different graphene content are shown in Figure 5. When 0.2 wt.% graphene was added into concrete, the resistivity of the concrete decreased significantly, and the conductivity increased. Comparing Figure 5a with Figure 5b, the fine floccules formed by graphene and cement hydration products have an obvious wrapping effect on the carbon fibers. Thus, the effective conductive contact area of carbon fiber and its surrounding cement hydration products and steel fiber was expanded. The defects of small contact area between carbon fiber and steel fiber and cement hydration products due to their large length-diameter ratio were repaired. The electrical conductivity was then enhanced to a certain extent.

When the graphene content increased to 0.4 wt.%, Figure 5c shows that the graphene flocs gradually increased, which could better encapsulate the carbon and steel fiber, enabling formation of a stable three-phase composite conductive network, enhancing the overall conductivity significantly.

As shown in Figure 5d, when the graphene content increased to 0.6 wt.%, the water absorption effect of graphene increased correspondingly, resulting in dry floccules formed by graphene and cementitious materials, shrinkage deformation, and slightly decreased connectivity with steel fiber and carbon fiber, creating small gaps. The effective contact area of carbon fiber, steel fiber, and conductive cementitious materials was slightly reduced, and the effect of resistivity improvement was not obvious.

When the content of graphene was further increased to 0.8 wt.% and 1 wt.%, it can be seen from Figure 5e,f that graphene absorbed a large amount of water due to its large specific surface area, resulting in obvious shrinkage deformation in the concrete. The formation of more holes and gaps increased the separation of carbon fiber and steel fiber, which was not conducive to the orderly formation and complete overlap of the conductive network. In addition, the additional holes and gaps increased the potential energy of the electronic transition during the electrification, and the resistivity increased significantly.

### 3.4. Snowmelt Test of Graphene Composite Conductive Concrete Slab

The snow melting process of the graphene composite conductive concrete is a process of converting electrical energy into thermal energy. When using the thermal effect of conductive concrete to melt snow, according to the law of conservation of energy, it is known that the heat generated by the conduction of graphene conductive concrete should be equal to the sum of the heat absorbed by the heating of concrete slab, the heat lost in concrete slab and air, and the heat absorbed by the heating and melting of snow. The relationship is expressed as:

(2) $$P\times \Delta t=mC_{P}\Delta_{T}+m_{i}C_{Pi}\Delta_{Ti}+Q_{L}m_{i}+Q_{s}$$

where *P*, electric heating power, W; ∆*_t_*, snowmelt time, s; *m*, quality of graphene conductive concrete, kg; *C_P_*, specific heat capacity of graphene conductive concrete, J/kg·K; ∆*_T_*, temperature change of concrete slab, K; *m_i_*, quality of snow, kg; *C_Pi_*, specific heat capacity of snow, J/kg·K; ∆*_Ti_*, average temperature change in snow during snow melting, K; *Q_L_*, melting heat of snow, J; *Q_S_*, heat loss during snow melting, J.

The energy utilization ratio of graphene composite conductive concrete is the ratio of heat absorbed by snow heating and melting to input electric energy:

(3) $$\phi =\frac{P\Delta t-mC_{P}\Delta t-Q_{s}}{P\Delta t}\times 100\%$$

After applying voltage at both ends of the graphene composite conductive concrete plate, the current flows in the low resistance area of the plate, and a large amount of Joule heat will be produced over time. The generated heat first increases the temperature inside the plate. According to the heat conduction effect, when there is a temperature gradient inside or between objects, there will be energy transferred from the high temperature region to the low temperature region. Therefore, part of the heat will remain in the interior of the plate and continue to heat, and the other part will transfer to the surface of the plate with low temperature, which will increase the surface temperature. When the temperature on the surface of graphene composite conductive concrete slab rises above 0 °C and exceeds the melting point of ice and snow, the heat absorbed by snow covering the surface of the slab begins to melt, to achieve the purpose of electrified snow melting.

#### 3.4.1. Effect of Different Electrode Spacings on Snowmelt Performance

**Figure 6:**
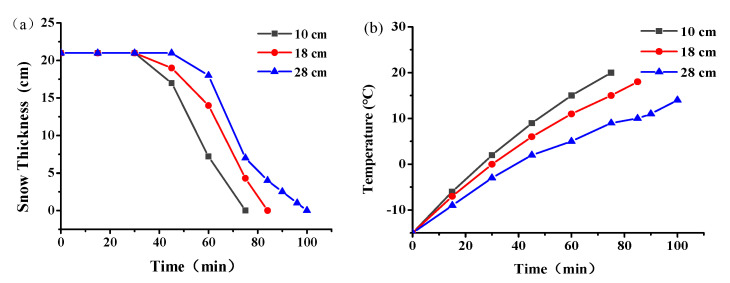
Snowmelt effect of graphene conductive concrete with different electrode spacing; (**a**) snow thickness (**b**) plate temperature rise.

The snowmelt effect of graphene composite conductive concrete slabs with different electrode spacing at 220 V and a snow thickness of 21 cm is shown in Figure 6. It can be seen from Figure 6a that the snowmelt effect of the graphene composite conductive concrete slab can be divided into four stages. In the first stage, the snow thickness remained basically unchanged with the increase of power over time. This is because in the early stage of power supply, the current heat inside the slab is mainly used to overcome the low temperature environment to work and increase the temperature of the slab. The current heat generated inside the concrete is mainly transmitted to the snow layer on the surface of the concrete through heat conduction and thermal radiation. However, since the overall temperature of the plate has not yet reached 0 °C (Figure 6b), which does not exceed the melting point of snow and ice, and the specific heat and dissolution heat of snow are larger than those of the graphene composite conductive concrete, the snow in the first stage has not yet begun to melt.

After the second stage was electrified for 35 min, the snow on the graphene composite conductive concrete slab began to melt under different electrode spacings. Figure 6b shows that with the conduction of electricity, the surface temperature of the plate exceeded 0 °C, and gradually exceeded the melting point of snow. The snow on the surface of the plate gradually absorbed heat and begins to melt. As the temperature on the surface of the plate increased to more than 0 °C, the heat transferred from the plate itself to the snow layer through thermal radiation gradually increased, which further promoted the melting of snow.

After a period of continuous power supply in the third stage, the snow melting rate increased. With the continuous conduction time, it can be seen from Figure 6b that the temperature on the surface of the plate gradually increased, but the temperature gradient gradually decreased. The energy absorbed by the plate heat storage was reduced relative to the previous period, and the heat transferred to the snow layer by the plate heat radiation gradually increased. In this stage, the total heat generated by electricity was basically unchanged, and the current heat generated by the plate distributed to the proportion of snow melting increases, thus accelerating the melting of snow. Therefore, the snowmelt rate of the third stage was faster than the initial snowmelt rate of the second stage.

In the fourth stage, the snow melting rate gradually decreased until the snow melting was completed. This is because with the decrease of snow thickness, the effect of wind speed on heat loss near the snow layer was more significant, which reduced the retention of heat in the snow and affected the energy saving of the snow layer. Therefore, in the fourth stage, the heat transferred to the snow layer was reduced, and the heat storage of the snow layer itself was also reduced, so the snow melting rate was weakened compared with that in the third stage.

**Figure 7:**
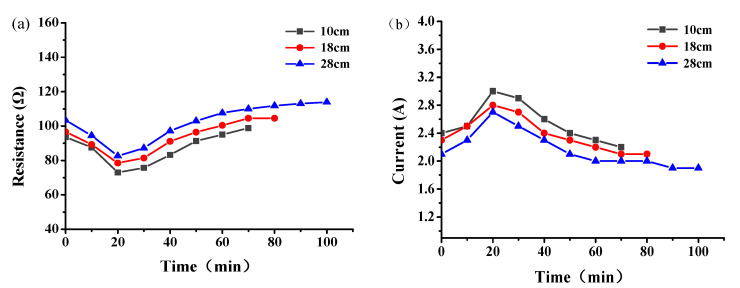
Resistance (**a**) and current (**b**) of graphene composite conductive concrete slabs with different electrode spacings.

The resistance and current of the graphene composite conductive concrete slab at different electrode spacings are shown in Figure 7. With the increase of snow melting time, the resistance of the plate first decreased and then increased (Figure 7a), while the current first increased and then decreased (Figure 7b), and both reached the extreme values at 20 min of snow melting. In addition, the resistance of the plate increased with the increase of electrode spacing, the passing current decreased, and the total time required for melting the same snow increased. According to the law of conservation of energy, the heat generated by the electric conduction of the conductive concrete should be equal to the sum of the heat absorbed by concrete heating, the heat absorbed by ice and snow heating and melting, and the loss of heat exchange between the plate and the surrounding environment. With the increase in electrode spacing, the energy loss of the current flowing from the positive electrode to the negative electrode in the plate increased, and the energy absorbed by the snow melting was constant. Therefore, it was necessary to extend the power-on time to further supply the energy needed for snow melting, and with the extension of the power-on time, the total energy consumption gradually increased.

**Figure 8:**
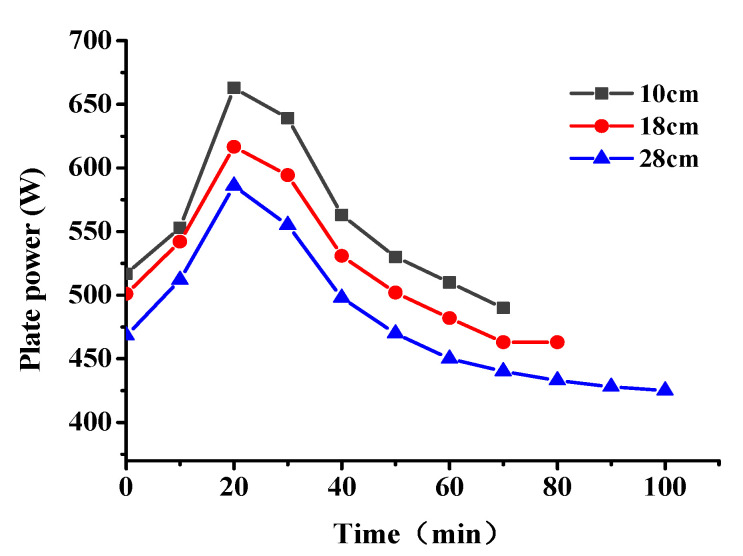
Input power of graphene composite conductive concrete plate with different electrode spacing.

The snow-melting power of the conductive concrete slab with different electrode spacing is shown in Figure 8. The snow-melting power of the plate increased with decrease in the electrode spacing. With the increase of snowmelt time, the power of the plate first increased and then decreased; the maximum power of 663 W was achieved after 20 min snowmelt. Combined with the temperature-resistance law, it can be seen that the resistance of the plate increased with increase of the plate temperature, and the heat production power and heat transfer efficiency show a decreasing trend; this phenomenon was more obvious with increase in the electrode spacing.

#### 3.4.2. Effect of Different Voltage on Snowmelt Performance

**Figure 9:**
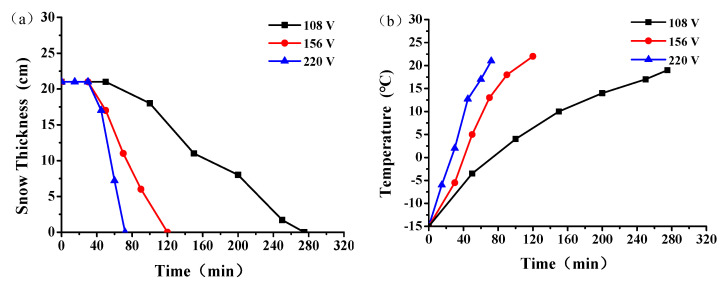
Snowmelt effect of graphene composite conductive concrete under different voltages. (**a**) snow thickness, (**b**) plate temperature rise.

The snow melting effect of the graphene multiphase conductive concrete slab under different energizing voltages when the electrode was spaced at 10 cm is shown in Figure 9, when the snow thickness was 21 cm. It can be seen from Figure 9a that when the external voltages were 108 V, 156 V, and 220 V, the snow melting process corresponded to that in Section 3.4.1, which can be divided into four stages. With the increase of input voltage, the snow-melting efficiency of the graphene composite conductive concrete slab was significantly enhanced. When the input voltage was 108 V, 156 V, and 220 V, the melting time of snow was 276 min, 120 min, and 72 min, respectively. In addition, Figure 9b shows that with the increase of input voltage, the temperature rise rate was significantly accelerated. When the input voltage was 108 V, 156 V, 220 V, the temperature rise rate was 0.06 °C/min, 0.18 °C/min, and 0.29 °C/min, respectively.

**Figure 10:**
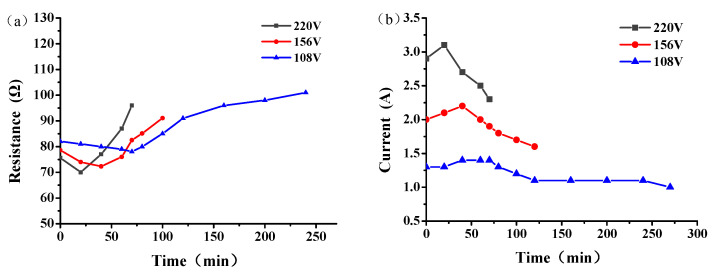
Resistance (**a**) and current (**b**) of graphene composite conductive concrete slabs under different voltages.

The resistance and current of the graphene composite conductive concrete plate under different voltages are shown in Figure 10. When the electrode spacing was 10 cm, with the increase of snow melting time, the resistance of the plate first decreased and then increased, while the current first increased first and then decreased. Under the same initial conditions, with the increase of the voltage, the snow melting time of the plate was shortened, and the energy consumption first decreased and then increased.

**Figure 11:**
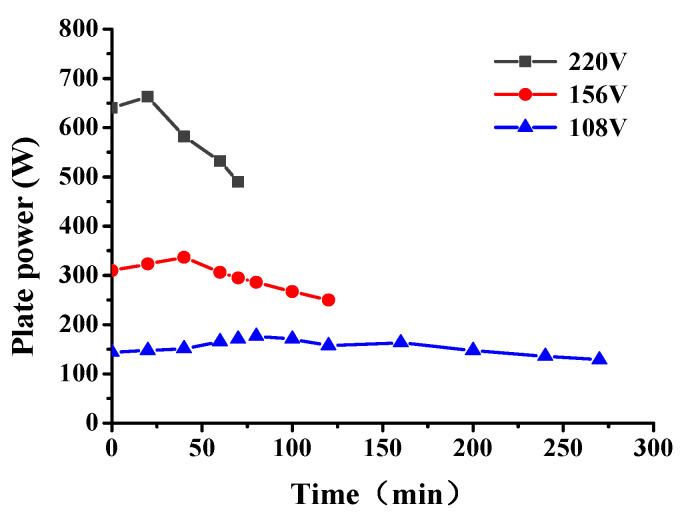
The input power of graphene composite conductive concrete slab changes with the on-voltage.

**Figure 12:**
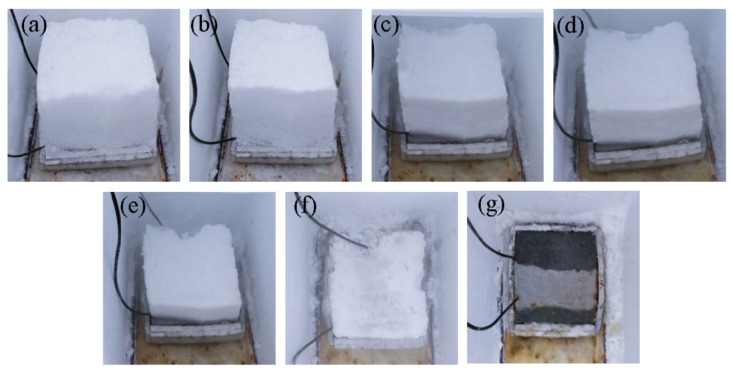
Real-time snowmelt diagram: (**a**) 0 min, (**b**) 20 min, (**c**) 40 min, (**d**) 60 min, (**e**) 80 min, (**f**) 100 min, (**g**) 120 min.

The input power of the graphene composite conductive concrete slabs at different voltages is shown in Figure 11. When the initial snow melting temperature was −15 °C, the environment was relatively harsh. When the electrified voltage was 108 V, the thermal power density of the plate was low, which was not enough to overcome the low temperature environment and the internal obstacles of the plate. The total energy absorbed by the plate heating from −15 °C to 0 °C and melting snow of 21 cm thickness can be predicted. When the heating power density is low, the required power duration will be prolonged and the total energy consumption will be increased. When the power supply voltage increases to a more appropriate value, the thermal power density of the plate just reaches the boundary value of the temperature rise of the plate to overcome the surrounding low temperature environment and internal obstacles, which effectively reduces the snow melting time and further reduces the energy loss. As the electrified voltage increased to 220 V, the power density of snowmelt was relatively surplus. Although the electrified time was short and the heat loss process was shortened at this time, the power of heat loss significantly increased. It can be seen from W = Pt that the overall heat energy loss was high at this time, 18% higher than that when the electrified voltage was 156 V. In summary, under the corresponding snow melting conditions, when the snow melting time and energy consumption are taken as the evaluation indexes, there was an optimal voltage of 156 V; at this time the board could melt 21 cm thick snow with a relatively low power in 2 h. The real-time snow melting effect is shown in Figure 12.

## 4. Conclusions

Three phase composite conductive concrete was prepared with multilayer graphene, carbon fiber, and steel fiber as conductive materials. The optimum content of graphene in conductive concrete was sought with resistivity as the criterion. The effects of electrode spacing and energizing voltage on snow melting efficiency were studied. The following conclusions can be drawn from the results of this study:

1. When the content of graphene was 0.4% of the cement mass, the mechanical properties and electrical conductivity of the conductive concrete were significantly improved. The compressive strength first increased and then decreased with the increase of graphene content and reached a maximum strength of 45.3 MPa when the content was 0.4%. The resistivity decreased with increase in graphene content. When the content reached 0.4%, the resistivity gradually stopped decreasing and showed an upward trend; the minimum resistivity was 12.66 Ω·m.
2. With increase in electrode spacing, the snow-melting efficiency and energy consumption of the graphene composite conductive concrete slab reduced. When the electrode spacing was 10 cm, snow of 21 cm thickness could be rapidly melted within 1.2 h, with a required average power density of 6.5 kW/m^2^ and energy consumption density of 7.8 kW·h/m^2^.
3. The snow melting power of the graphene conductive concrete increased with increase in the electrified voltage, but the overall snow melting energy consumption first decreased and then increased with increase in the voltage. From an economic point of view, when the voltage was 156 V, the overall snow melting energy consumption was optimal.
4. With 21 cm snow thickness formed in 24 h, simulating the conditions of a heavy snowstorm, the graphene composite conductive concrete slab prepared in this experiment could quickly melt the snow in 2 h with electrode spacing of 10 cm and an input voltage of 156 V, without affecting road traffic. The average power density was 3.3 kW/m^2^ and the energy consumption density was 6.6 kW·h/m^2^.

## Figures and Tables

**Table 1:** Performance parameters of graphene.

Diameter (μm)	Thickness (nm)	Tier Number	Single Layer Rate	Purity	Specific Surface Area (m^2^/g)
10–50	3.4–7.0	6–10	30%	>95%	450–550

**Table 2:** Performance parameters of PAN-based carbon fiber.

Length (mm)	Monofilament Diameter (μm)	Carbon Content	Density (g/cm^3^)	Tensile Strength (MPa)	Tensile Modulus(GPa)	Volume Resistivity (Ω·cm)	Length to Diameter Ratio
6	7.0–10	≥95%	1.6–1.76	3.6–3.8	220	1.5 × 10^−3^	600–857

**Table 3:** Performance parameters of steel fiber.

Length (mm)	Monofilament Diameter (μm)	Density (g/cm^3^)	Tensile Strength (GPa)	Volume resistivity (Ω·cm)	Length to Diameter Ratio
13	200	2	2.85	1.5 × 10^−5^	65

**Table 4:** Proportion of graphene composite conductive concrete (kg/m^3^).

Cement	Coarse Aggregate	Sand	Water	Water Reducing Admixture	Carbon Fiber	Steel Fiber	Graphene	Dispersing Agent
450	975	766	198	2.7	1.8	20	0	0
							0.9	1.8
							1.8	3.6
							2.7	5.4
							3.6	7.2
							4.5	9.0

**Table 5:** Compressive strength of graphene composite conductive concrete with different graphene content at 28 d age.

Graphene content/wt.%	0	0.2	0.4	0.6	0.8	1.0
Compressive strength/MPa	42.5	44.1	45.3	40.7	39.1	37.6

## Data Availability

The data used to support the findings of this study are available from the corresponding author upon request.
